# Transcription factors in late megakaryopoiesis and related platelet disorders

**DOI:** 10.1111/jth.12131

**Published:** 2013-04-11

**Authors:** M R Tijssen, C Ghevaert

**Affiliations:** * Department of Haematology, University of CambridgeUK; † Department of Haematology, University of Cambridge, and NHS Blood and TransplantCambridge, UK

**Keywords:** megakaryocyte, platelet formation, thrombocytopenia, transcription factor

## Abstract

Cell type-specific transcription factors regulate the repertoire of genes expressed in a cell and thereby determine its phenotype. The differentiation of megakaryocytes, the platelet progenitors, from hematopoietic stem cells is a well-known process that can be mimicked in culture. However, the efficient formation of platelets in culture remains a challenge. Platelet formation is a complicated process including megakaryocyte maturation, platelet assembly and platelet shedding. We hypothesize that a better understanding of the transcriptional regulation of this process will allow us to influence it such that sufficient numbers of platelets can be produced for clinical applications. After an introduction to gene regulation and platelet formation, this review summarizes the current knowledge of the regulation of platelet formation by the transcription factors EVI1, GATA1, FLI1, NFE2, RUNX1, SRF and its co-factor MKL1, and TAL1. Also covered is how some platelet disorders including myeloproliferative neoplasms, result from disturbances of the transcriptional regulation. These disorders give us invaluable insights into the crucial role these transcription factors play in platelet formation. Finally, there is discussion of how a better understanding of these processes will be needed to allow for efficient production of platelets *in vitro*.

## Introduction

Platelets are the second most abundant cell in the blood. They monitor blood vessels for damage. Upon injury they initiate blood clotting and contribute to vessel wall repair. On the flip side of the coin platelets cause thrombosis when too many and/or hyperactive platelets cause vessel occlusion, leading to heart attacks or strokes. Each day about 10^11^ platelets are formed from their precursor cells, the megakaryocytes (MKs).

The hematopoietic system and its lineage decisions have been characterized in some detail. Transcription factors are key regulators of these decisions. Over the last decade a lot of progress has been made in identifying the factors involved in the differentiation of the bipotent megakaryocyte-erythroid progenitor (MEP) from hematopoietic stem cells (HSCs) in the bone marrow (BM). Also, factors involved in the lineage bifurcation into committed early erythroid or megakaryocytic cells have been identified, making it possible to model MK/erythroid differentiation 1. Changes in the transcriptional program responsible for maturation of early MKs into cells that shed platelets are less clear. This review summarizes our current knowledge of the transcriptional regulation of the final stages of MK maturation and platelet formation. We discuss how dysregulation may lead to platelet disorders. Finally, fields of research that hold promise for improving our understanding of MK maturation and platelet formation are touched upon.

## Gene regulation

Each cell of the body contains the same genetic information. Cell function and identity are defined by the genes that each cell expresses. Expression of actively transcribed genes is generally initiated by binding of ubiquitously expressed general transcription factors to the TATA sequence in the promoters of these genes. This leads to the recruitment of the transcription machinery consisting of other regulatory co-factors and RNA polymerase 2. Specificity is introduced by the tight regulation of the expression of a repertoire of cell type-specific transcription factors. These transcription factors recognize certain sequences in the DNA called motifs. These motifs are usually not long and appear many times in the DNA. Whether a transcription factor will bind to its motif depends on the accessibility of the DNA. To accommodate the large eukaryotic genome in the nucleus the DNA is packed into chromatin. It is organized in nucleosomes by histone proteins and repeating units of nucleosomes make up the chromatin. The cell uses modifications of the histone proteins to regulate the tightness of the packaging and thereby DNA access to transcription factors and the transcriptional machinery. Interestingly, these modifications are inherited by daughter cells and are thus one of the ways to regulate cell type-specificity 3.

**Figure 1:**
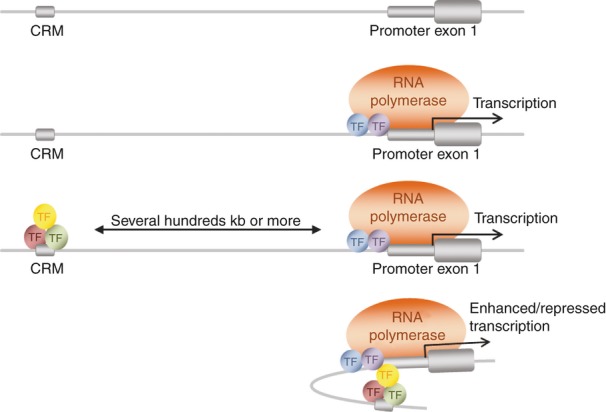
Schematic representation of how a cis-regulatory module can enhance transcription.

Complexes of different transcription factors can bind a single DNA element, inducing transciptional activation or repression 4 (Fig. 1). Depending on the constituents of the complex it may act to activate or repress transcription. The crucial role of transcription factors in lineage specification from HSC to MK and subsequent MK maturation is well documented 5, but the exact role that these transcription factors may play in making MKs specifically capable of platelet formation is not as clear. Some evidence can, however, be gleaned from existing data and is discussed below after introducing some essential cell biology concepts that are particular to megakaryocytes and platelet formation.

The specificity for spatio-temporal regulation of gene expression is acquired by combinatorial binding of multiple transcription factors 6. Transcription factors and co-activators bind at the promoter of a gene proximal to the transcription start site. Recruitment of RNA polymerase will initiate transcription. Sets of transcription factors binding on a distal DNA element are called *cis*-regulatory modules (CRMs). CRMs can be located several hundreds of kilobases (kb) or even more away from the promoter and still function as enhancers/repressors 7. Looping of the DNA allows the CRM to interact with the factors bound at the promoter and to considerably enhance/repress transcription 8–9.

## Megakaryopoiesis and platelet formation

### Signals that promote megakaryopoiesis

The main driver of MK differentiation is the binding of thrombopoietin (TPO) to its receptor MPL 10. The subsequent dimerization of the receptor induces the autophosphorylation of the janus kinase 2 (JAK2). JAK2 phosphorylates a number of downstream substrates, leading to the activation of multiple signaling pathways, including mitogen-activated protein kinases (MAPK), phosphoinositol-3 kinase (PI3K) and signal transducers and activators of transcription (STATs). The ultimate effect of the activation of these signaling pathways is induction and repression of gene expression and MK differentiation.

Besides TPO, other cytokines, chemokines and extracellular matrix proteins influence megakaryopoiesis 11–12. Noteworthy is the residual level of platelets (about 15%) in *Tpo* or *Mpl* knock-out mice, probably driven by the interaction between the MK progenitor and the BM endothelial niche 13.

### Platelet formation

#### Platelet assembly in proplatelets and platelet shedding

Maturation of MKs is accompanied by successive rounds of DNA replication without cytokinesis (endomitosis), resulting in large polyploid cells with a lobulated nucleus. Concurrently, the MK matures into a cell that contains all the machinery necessary for platelet function. Secretion granules appear in the cytosol, which, in addition to many constituents found in other cells, also contain platelet-specific proteins that promote coagulation and vessel repair. MK maturation is also accompanied by the expression of cell surface receptors that allow platelet adhesion and aggregation.

These cytoplasmic and membrane constituents ultimately need to be packaged into the small anucleate platelets before they are shed into the circulation. A widely accepted model for platelet assembly and shedding is the proplatelet model. BM MKs develop multiple branched extensions that protrude into the marrow sinusoids, where their terminal buds are released into the circulation 14. The extensive network of demarcation membrane formed during MK maturation may serve as a reservoir of membrane for proplatelet formation 15. Dynamic organization of tubulin and the sliding of microtubules past one another are essential to the elongation of the proplatelet branches and the accumulation of granules in the buds 16–17. In keeping with this observation, disruption of the MK-specific TUBB1 (β1-tubulin) in mice, dogs and human patients leads to macrothrombocytopenia 18,19. Actin and myosin control branching of the proplatelet elongations and MYH9 (myosin IIa) alongside its regulator RHOA restrains platelet formation. Consequently, mutations that reduce myosin IIa activity, such as seen in the MYH9-related May-Hegglin syndrome, lead to inappropriate platelet shedding, causing macrothrombocytopenia 21–22. Recently, the ITIM-containing receptor G6b-B was implicated in proplatelet formation. Knock-down of G6b-B results in macrothrombocytopenia which was shown to result, at least partly, from aberrant integrin signaling disrupting cytoskeletal remodeling 23.

Release of MK fragments with proplatelet characteristics into the circulation has been observed *in vivo* in mice 24. However, as the actual release of platelets from these proplatelets has not been observed as such, some claim that proplatelet formation is an artefact and propose an alternative hypothesis where platelets are formed by cytoplasmic fragmentation of the MK 25.

#### Triggers for platelet assembly and shedding

As the process of platelet formation is still debated, so is the trigger for this process. Although the role of TPO in MK maturation is undisputed, TPO seems dispensable for platelet formation and maybe even inhibits this process 26–27. Removal of growth factors commonly triggers cell death. Therefore, the dispensability of TPO may be related to the fact that proplatelet formation has been described as a form of compartmentalized caspase-dependent cell death. Over-expression of anti-apoptotic molecules (chiefly BCL2 and BCL2L1 [BclxL]) or reduced expression of proapoptotic members of the BCL2 family (BCL2L11 [Bim]) reduces platelet formation by MKs 28,29, as does pharmacological inhibition of caspases 31–32. A recent study confirmed the central role played by BCL2L1 in platelet release from mature MKs, although in this particular study, mice with combined deletion of both proapoptotic BAK1 and BAX proteins showed no alteration of platelet formation 33. Also, a lack of caspase-9 does not affect steady-state platelet formation 34. These two studies from the same group suggest that the intrinsic (mitochondrial) apoptosis pathway is dispensable for platelet formation.

Location of the MK and interaction with its environment seems to be crucial for platelet formation. It is known that the relocation of immature MKs from the osteoblastic niche to the endothelial niche driven by chemotactic agents is essential for maturation and platelet production 12. Here, contact with bone marrow endothelial cells (BMECs) induces further maturation and platelet formation 35. The chemokine CXCL12 (SDF1) and growth factor FGF4 promote both MK migration and interaction with the BMECs, thereby promoting platelet production 13. Interestingly, this interaction is enhanced by inflammatory cytokines, such as IL1B (interleukin-1 beta) 33–34. Furthermore, stimulation of the pro-inflammatory VEGFR1 pathway leads to an up-regulation of the CXCL12 receptor CXCR4 and increased *in vivo* platelet formation 36. These findings suggest that increased interaction between MKs and stimulated endothelium is responsible for the thrombocytosis often observed in inflammation 37. It is also clear that in diseases where MK migration is impaired, such as Wiskott-Aldrich syndrome (WAS) where actin polymerisation is disturbed, have impaired platelet formation 38. The same is true for thrombocytopenia induced by compounds such as Dasatinib that decrease MK migration 39.

In addition to this direct MK-to-endothelial cell contact, extracellular matrix proteins also influence proplatelet formation. Type I collagen inhibits proplatelet formation through integrin αIIβI 40. Fibrinogen, on the other hand, promotes proplatelet formation through glycoprotein IIB/IIIA (GPIIB/IIIA; integrin αIIbβ3). Mutations causing constitutive GPIIB/IIIA activation interfere with proplatelet formation and lead to the production of very large platelets (macrothrombocytopenia) 41–42. There is also evidence that von Willebrand factor (VWF) binding to the GPIB/V/IX complex regulates proplatelet formation, which may be the reason for the macrothrombocytopenia observed in patients with Bernard-Soulier syndrome who suffer from a defect in the GPIB/V/IX complex 43. Platelet production seems therefore under the control of several adhesive interactions between the MK, the BM vasculature and the extracellular matrix. However, these do trigger the protrusion of the proplatelet extensions into the sinusoids. For this, one would expect the presence of a compelling factor in the serum, which could be sphingosine 1-phosphate 44–45.

### Uncoupling of polyploidization from terminal differentiation and proplatelet formation

Surprisingly, proplatelet formation is not strictly within the remit of the higher ploidy MKs. Both *in vivo* and in culture 2N or 4N MKs form proplatelets 46,47 and platelet numbers are similar in mouse backgrounds with different BM MK ploidy 49. Therefore, although some cytoskeletal (TUBB1, MYH9, RAC1 and RAP1B), transmembrane (GPIIIA, GP1BA and B) and signaling proteins (LAT and SRC family kinases) known to be involved in proplatelet formation are slightly more abundant in high ploidy MKs, this appears not to affect platelet shedding. However, instead of having an effect on its ability to produce platelets, the ploidy level of an individual MK might influence protein content. Platelets originating from high ploidy MKs might be more easily activated than platelets generated from MKs with a lower ploidy 50.

It has been hypothesized that polyploidization is required to meet the MK's vast need for protein synthesis and cell growth. This is supported by a study by Raslova *et al*. 51 showing that all alleles of a series of MK-specific genes in cells ranging from 4N to 32N are functional and not epigenetically silenced. However, a subsequent study by the same authors showed that multiple genes involved in platelet formation and DNA proliferation are regulated differently depending on ploidy levels 52. Thus, specific gene regulatory processes are at work at different levels of ploidy to ensure the MK develops into a functional platelet-producing cell. Indeed, murine MKs with high ploidy have down-regulated genes involved in DNA replication and up-regulated genes involved in cytoskeletal dynamics, cell migration, G-protein signaling and platelet function 53.

In keeping with the idea that platelet shedding is not necessarily coupled with ploidy, genes regulating platelet production, such as NFE2 (nuclear factor [erythroid-derived 2], discussed below), are not involved in the regulation of polyploidization 54, and inversely, genes that increase MK ploidy, such as CCND3 (cyclin D3), do not modify the platelet count 55.

## Transcription factors implicated in late megakaryocyte differentiation and related diseases

**Figure 2:**
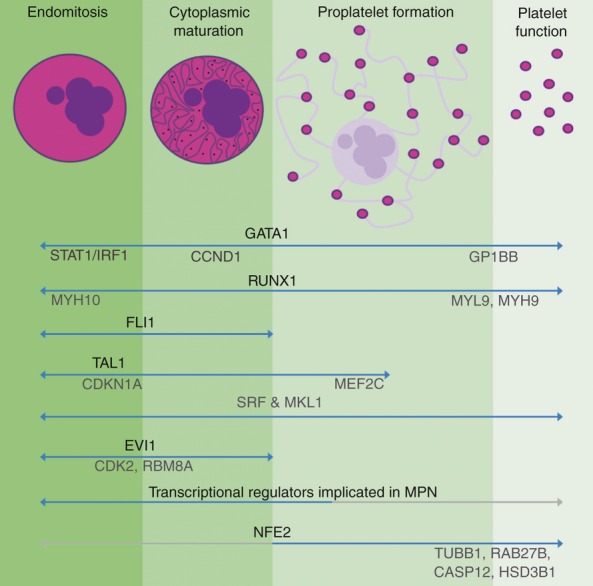
Summary of the transcription factors and downstream targets implicated in different stages of thrombopoiesis.

Specific transcription factors implicated in late MK differentiation and abnormalities causing disease are summarized in Fig. 2. Transcription factors that have been implicated in the cell fate decision from stem cell to megakaryocyte or in the early phase of megakaryopoiesis only will not be discussed.

Late MK differentiation involves endomitosis, cytoplasmic maturation and proplatelet formation. Depicted are the transcription factors (black) shown to play a role in these processes and, if identified, their downstream targets (grey) performing this role. Some of these also affect platelet function. The arrow indicates in which stages the transcription factors have been implicated (blue, strong evidence; grey, weak evidence).

### GATA1/2 and Bernard-Soulier syndrome, X-linked thrombocytopenia and acute megakaryoblastic leukemia

GATA1 (GATA-binding factor 1) and GATA2 are zinc-finger transcription factors. Both bind to a common co-factor ZFPM1 (FOG1; friend of GATA 1). GATA1 is highly expressed in erythroid cells, mast cells and MKs. Although GATA2 plays a role in the maintenance of early multipotent stages of hematopoiesis, it is also expressed in MKs and there are indications that GATA2 may have an overlapping function with GATA1 56. A lack of GATA1 in the MK lineage leads to decreased polyploidization and a lack of cytoplasmic maturation 57,58. Restoration of the levels of genes targeted by GATA1, such as CCND1 (cyclin D1), increases MK size and DNA content in *Gata1* knock-out MKs, but does not rescue expression of late MK genes or proplatelet formation 59. In contrast, ectopic expression of STAT1 (or its downstream effector IRF1) not only induces polyploidization, but also enhanced expression of a subset of platelet-specific genes. In keeping with this concept, a point mutation in a GATA-binding site in the *GP1BB* promoter proximal region causes a form of Bernard-Soulier syndrome 60, illustrating the link between GATA1 and expression of a typical marker of mature MKs.

Mutations in *GATA1* are causative for a number of human disorders, further illustrating its central role in megakaryopoiesis 61. In X-linked thrombocytopenia, missense mutations that decrease its affinity for ZFPM1 or its DNA-binding ability not only affect the number but also the maturation of MKs. In addition, the few platelets that are eventually produced have an abnormal size and contain few alpha granules. GATA1 is also implicated in the development of acute megakaryoblastic leukemia (AMKL). AMKL can be separated into three subtypes: pediatric AMKL, either associated with Down Syndrome (DS-AMKL) or not, (non-DS-AMKL) and adult AMKL. Apart from having a very different clinical prognosis (DS-AMKL has generally a much better prognosis than the other two), each differs in its genetic abnormality. DS-AMKL, in particular, is associated with somatic mutations in the *GATA1* gene, which universally leads to the expression of a truncated form called GATA1s. GATA1s overlaps in its function with GATA1 in that it allows for commitment to the MK lineage, but does not allow full maturation of the MK. This leads to marked expansion of MK progenitors. It is believed that this is the mechanism that leads to a pre-leukemic transient myeloproliferative disorder (TMD) present in 4–10% of newborns with DS. TMD is characterized by an expansion of immature Mks, but subsequently often undergoes spontaneous remission. The development of DS-AMKL requires additional hits to the trisomy 21 and GATA1s mutations. For example, over-expression of ETS family transcription factors such as ERG (a transcription factor of early megakaryopoiesis 62) can immortalize GATA1s cells, but not cells expressing full-length GATA1 63.

### RUNX1 and familial platelet disorder with propensity to develop acute myeloid leukemia

RUNX1 (AML1) is a member of the RUNT family of transcription factors and together with its cofactor CBFB (core-binding factor, beta subunit) represents the most common mutational target in human acute leukemia. RUNX1's essential role in HSCs is illustrated by the fact that emergence of all definitive hemopoiesis out of the aorto-gonadal-mesonephros during embryogenesis is prevented in mice that completely lack RUNX1. Conditional knock-out studies also show a fundamental role for RUNX1 in megakaryopoiesis, with a marked decrease in polyploidization and cytoplasmic development of MKs, similar to what is observed for *Gata1*. The complex role of RUNX1 in MK differentiation is further illustrated by the autosomal dominant human syndrome familial platelet disorder with propensity to develop acute myeloid leukemia (FDP/AML), in which germline heterozygote *RUNX1* mutations lead not only to thrombocytopenia, but also to impaired platelet function. In addition, FDP/AML patients also carry a high risk of development of myelodysplasia and leukemia. Platelets from these patients have abnormal expression of, amongst others, the TPO receptor 64 and MYL9 (myosin light chain 9) 65. In MKs cultured from these patients’ stem cells, expression of non-muscle myosin is perturbed, with persistent expression of MYH10 (non-muscle myosin IIb) and decreased expression of MYL9 and MYH9 (non-muscle myosin IIa) 66. Silencing of MYH10 by RUNX1 contributes to the transition from mitosis to endomitosis, as in immature MKs MYH10 specifically localizes to the contractile ring separating the cells 67. Thus, RUNX1 regulates constituents of the MK and platelet cytoskeleton and thereby late megakaryopoiesis and platelet formation.

### FLI1 and Paris-Trousseau syndrome

Friend leukemia virus integration 1 (FLI1) and another member of the family of ETS transcription factors, GABPA (GA binding protein transcription factor, alpha subunit), seem to act in tandem in MKs. As MK maturation progresses, the ratio of FLI1/GABPA increases. In keeping with this observation, GABPA regulates expression of early MK genes (including GPIIB and the TPO receptor MPL) whilst FLI1 binds to both early and late (GPIBA, GPIX, PF4 [platelet factor 4]) MK genes 68. This is clearly illustrated by the Paris-Trousseau syndrome, an inherited disorder associated with 11q chromosome deletion with thrombocytopenia and an increased tendency to bleed. FLI1 hemizygous loss due to a deletion underlies the disease. Patients show a maturation block with microMKs on BM smears and typical abnormal granule formation 69–70.

### Tal1

Conditional knock-out studies of TAL1 (T-cell acute lymphocytic leukemia 1, SCL) in the hemopoietic lineage have shown a specific decrease of red cell and MK production 71. Knock down of TAL1 translates into a lack of proliferation, polyploidization and cytoplasmic maturation and reduced platelet numbers but not platelet function in MKs 72. One of the targets of TAL1 is the cell cycle regulator CDKN1A (cyclin-dependent kinase inhibitor 1A, p21), which is over-expressed upon knock down of TAL1 expression. Crucially, knock down of CDKN1A in TAL1-mutant MKs restores the endomitotic cell-cycle progression, but only partially restores the cytoplasmic maturation necessary for the production of fully functional platelets. Thus other targets of TAL1 are also responsible for the defects in these late stages of MK maturation. One of these targets may be MEF2C (myocyte enhancer factor 2C), as mice lacking MEF2C in the hemopoietic lineage have reduced numbers of platelets with larger size and abnormal shape and granularity 73.

### SRF and MKL1 in megakaryocytic leukemia

Serum response factor (SRF) is a MADS-box transcription factor regulating growth factor-inducible genes and genes controlling cytoskeleton structures involved in cell spreading, adhesion and migration. Its role in megakaryopoiesis was identified when its co-factor MKL1 (megakaryoblastic leukemia [translocation] 1) was found to be involved in the *t*(1;22) translocation found in MK leukemias. SRF deficiency leads to a drop in platelet count accompanied by an increase in the BM content of MK progenitors, similar to that observed in NFE2 and FLI1-deficient animals 74. As this study focuses on the effect on stem cells and not MKs, it suggests that SRF acts mainly through cell-matrix interactions and integrin signaling, which may be of importance for retention of MKs in the endothelial niche of the BM and platelet formation 74. It was recently shown in double knock-out mice that MKL1 and its homologue MKL2 are both critical for MK maturation and platelet formation as well as function and that they exert these effects, at least partly, in an SRF-independent manner 75.

### EVI1 in thrombocytopenia with absent radii and acute myeloid leukemia

The *MECOM* locus on chromosome 3, encoding EVI1 (ecotropic virus integration site 1), is implicated in 4–6% of all AML cases. Interestingly, the so-called 3q21q26 syndrome leukemias present with particular dysmorphic MKs and an elevated platelet count. This is thought to be caused by inhibition of CDK2 (cyclin A dependent kinase inhibitor 2) expression mediated by the abnormal expression of EVI1 76.

EVI1 is expressed in hematopoietic progenitor cells, MKs and platelets. Ectopic expression in UT-7/GM cells changes these cells into polynuclear large cells that express PF4 77. Knock down of EVI1 in K562 cells reduces ITGA2B and ITGB3 expression after 12-O-tetradecanoylphorbol 13-acetate (TPA) treatment 78.

Additional evidence for the role of EVI1 in megakaryopoiesis is supported by a recent study showing that a mutation, creating an EVI1 binding site, discovered by exome sequencing in the promoter of the *RBM8A* (RNA binding motif protein 8A) gene can underlie the thrombocytopenia with absent radii (TAR) syndrome 79. Exome sequencing is aimed at reading the genetic code of an individual for all known coding regions across the genome (2% of the whole genome content). However, it also includes some of the non-coding ‘overhangs’ either side of the coding region, such as the 5'UTR (untranslated region). In TAR patients, a single nucleotide polymorphism (SNP) was discovered in the 5'UTR of *RBM8A*. The SNP increases binding of EVI1 and leads to a reduction of transcription of *RBM8A* and the encoded protein. TAR patients have low numbers of MKs in the BM that seem to have a maturation defect 80. In addition, there is some evidence that platelet function may be abnormal in these patients 81,82. Therefore, one might speculate that EVI1 regulation of RBM8A is crucial for late MK differentiation.

### Transcriptional regulators implicated in myeloproliferative neoplasms

The discovery by different groups in 2005 of a JAK2V617F mutation present in 50% of patients with essential thrombocythemia (ET), 60% of patients with myelofibrosis (MF) and over 90% of patients with polycythemia vera (PV) emphasized the importance of the JAK2 signaling pathway in MK growth and the production of platelets. The main downstream effectors of JAK2 are the STATs and the MAPK and the PI3K pathway, all of which ultimately regulate transcription.

There is evidence that not only MK growth, but also the last steps of maturation are altered in the above-mentioned myeloproliferative neoplasms (MPNs). One study showed that culture-derived MKs from patients with ET had an increased ability to form proplatelets and that the number of proplatelet-forming MKs in culture correlated with the platelet count in the patient from whom the MKs were derived 84. This has also been observed in a knock-in JAK2V617F mouse model of ET 85. A more indirect confirmation that JAK2V617F not only increases numbers but also influences cell biology is the observation that in patients with JAK2V617F mutations thrombosis in specific sites such as splenic veins can precede the development of overt overproduction of blood cells 86–87.

What is not clear at this stage is through which of the transcription factors JAK2V617F influences the maturing MKs. STAT3 analysis of a small group of ET patients showed that in about half of the patients, there was a STAT3 hyperactivation, but interestingly this did not correlate with the presence of the JAK2 mutation. In support of the central role played by STAT3 in MPNs, forced expression of RUNX1/MDS1/EVI1 in the BM of mice leads to an ET phenotype with an elevated number of dysplastic platelets with anisocytosis, degranulation and giant size 88. Although the RUNX1/MDS1/EVI1-positive mice did not harbour *Jak2* mutations, significantly higher levels of activated STAT3 were found in the BM as the complex of the three transcription factors binds to the *Stat3* promoter, inducing its expression.

It is well known that the MAPKs play a central role in megakaryopoiesis 89. Activation of the MAPKs has been documented downstream of JAK2V617F 90, and has also been seen in MPNs in the absence of the JAK2V617F mutation, either in the presence of the TPO receptor mutation W515L/K 91 or the KANK1-PDGFRβ fusion 92, in all cases causing a marked increase in the platelet count. FLT3-mediated MAPK activation also controls the abnormal megakaryopoiesis seen in MF 93.

Finally, JAK2V617F also activates the PI3K pathway, which can in turn exert a transcriptional effect through mammalian target of rapamycin (mTOR). There is ample evidence for a role of mTOR in megakaryopoiesis 94,95. Whilst to date its role in the context of MPNs is yet to be clarified, inhibition of mTOR seems to have a clinical effect in MF patients 97.

### NFE2 and MPNs

NFE2 is expressed in hematopoietic progenitor cells as well as in the myeloid, erythroid and MK lineages 98. NFE2 knock-out mice completely lack circulating platelets. This is despite an apparent increased number of MKs in the BM 54. It was therefore proposed that NFE2 acts as a regulator of proplatelet formation by promoting the final stage of maturation of MKs to the point where they are capable of platelet shedding. NFE2-deficient MKs can be grown *in vitro* in response to TPO, but are unusually large and have a disorganized demarcation membrane, and granules are small and sparse, indicating a late maturation block 99.

When expressed ectopically in BM cells, NFE2 also influences earlier stages of MK differentiation and allegedly enhances *in vivo* platelet production. However, transplantation of NFE2 over-expressing cells only accelerated platelet production and did not lead to an increase in the maximum level or total number of platelets detected in recipient blood 100. Over-expression of the transcription factor NFE2 (supposedly downstream of RUNX1) has also been reported in patients with all three myeloproliferative subtypes, independent of the presence or absence of the JAK2V617F mutation 101–102.

The apparent role of NFE2 in proplatelet formation might be explained through the function of some of its direct transcriptional targets, such as TUBB1 103, RAB27b 104, CASP12 (caspase 12) 105 and HSD3B1 (3-beta-hydroxysteroid dehydrogenase) 106. TUBB1 knock-out mice have thrombocytopenia with spherical platelets 107 and a mutation in TUBB1 has been identified in a patient with congenital macrothrombocytopenia 20. Mice with deficient Rab signaling have macrothrombocytopenia with few granules and abnormal MK morphology and RAB27B may coordinate granule transport during proplatelet formation 104. CASP12 null platelets have a defect in GPIIB/IIIA and, similarly, NFE2 null MKs fail to bind to fibrinogen in response to platelet agonists, which is indicative of a defect in the signaling leading to activation of GPIIB/IIIA 108. Finally, HSD3B1 (an enzyme implicated in estrogen metablolism) rescues proplatelet formation in NFE2 null MKs 106. The authors conclude that MKs may secrete autocrine estradiol that regulates proplatelet formation. Additional studies have shown that estrogens can induce MK differentiation 109–110, but to our knowledge this has not been successfully applied in an attempt to increase *in vitro* platelet formation.

## Interactions between the transcription factors involved in late megakaryopoiesis

Although it is clear that the transcription factors described above play a role in MK differentiation, the level of complexity regarding how they regulate gene expression is way beyond their individual function. Several of the transcription factors discussed above have been shown to interact with each other to regulate megakaryopoiesis, possibly in a linear hierarchy. For example, GATA1, GATA2 and TAL1 have been shown to regulate NFE2 111–112. Transcription factor biology has to be understood in the context of networks where each transcription factor will bind with a series of partners that will influence not only its DNA binding characteristics, but also its effect on transcription (i.e. activation or repression). For example, RUNX1 interacts with the mSin3A corepressor complex on the MPL promoter in hemopoietic stem and progenitor cells, while it forms a complex with a transcription activator EP300 (E1A binding protein p300) on the same promoter in MKs 113. Furthermore, TAL1 and associated proteins distinguish active from repressive GATA transcription factor complexes 114. In addition, transcription factors regulate not only expression of other partners with which they co-operate and physically associate, but also their own expression levels.

The advent of new generation sequencing and techniques such as chromatin immunoprecipitation (ChIP) allow us to identify where transcription factors bind in the genome. As transcription factor binding tends to cluster on sites that are critical for gene regulation, looking at multiple transcription factors simultaneously greatly improves the ability to infer biological relevance from a binding event. In a previously published study, we looked at simultaneous binding of GATA1, GATA2, RUNX1, FLI1 and TAL1 in primary human MKs 115. In keeping with the existence of transcription factor networks that collaborate to regulate gene expression, simultaneous binding of all five in a given genomic region was particularly enriched (i.e. it occurred far more than would be expected by chance). The five transcription factor binding sites identified 151 ‘candidate’ genes, some of which are already known to play a key role in megakaryopoiesis, whilst others are not. If one envisions a temporal hierarchy of transcription factors it is likely that, amongst the list of genes for which expression is putatively controlled by these five transcription factors, there are proteins that are crucial in particular for the latter stages of MK maturation, including platelet formation. This is illustrated by the fact that, for seven out of nine genes selected from the 151 target genes and not previously known to play a role in MKs, there was a clear thrombocyte phenotype upon morpholino knock down in zebrafish. Just as the genes identified in the study described above are now the subject of ongoing research, regulators of the very late stages of thrombopoiesis, in particular of the proplatelet formation process, will potentially be revealed by studies centred on additional genes that are controlled by transcription factors that have got a clear effect on proplatelet formation (such as NFE2).

Undoubtedly, microRNAs, epigenetic mechanisms and post-translational phenomena play an additional key role in the eventual expression of a given protein besides transcription factors. Detailed discussion of this additional layer of protein expression regulation in the context of the MK falls outside the remit of this review. It is, however, interesting to point out that the level of transcription factors involved in late MK differentiation and platelet formation, such as GATA1, FOG1, FLI1, TAL1, RUNX1 and NFE2, does not increase with polyploidization, whereas transcripts of their target genes are up-regulated 52. This may be due to several non-mutually exclusive mechanisms: translational or post-translational regulations or changes.

## Future perspectives

The study of the difference between fetal and adult megakaryopoiesis and platelet formation may uncover other gene regulatory mechanisms to add to our current knowledge. Fetal platelets are extremely large, with a diameter 1.6 times larger than adult platelets, and contain a large amount of RNA. This may be due to differences in gene expression regulation caused by differential expression of key transcription factors (such as GATA1) 96. Understanding the differences in fetal and adult thrombopoiesis has direct clinical implications. The lag time of platelet recovery following cord blood transplantation in comparison with transplant with stem cells derived from adult donors is a prime example of the consequence of the difference between neonatal and adult megakaryopoiesis. The progressive improvement of the platelet count in TAR patients after their first years of life is another example of the potential differential gene regulation in MKs in the fetus/neonate versus the adult, especially taken in the context that the genetic mutation responsible for the syndrome lies in a transcription factor binding site.

Ultimately, the knowledge of how transcriptional regulation affects megakaryopoiesis and platelet formation will be invaluable and relevant to clinical care. TAR and GATA binding-related Bernard-Soulier syndrome may be only two of a series of inherited disorders where the causative mutation lies not in a coding, but in a regulatory region of the genome. Understanding how these genetic variants can lead to disease will further inform our knowledge of how MK maturation is controlled at the transcriptional level. The same reasoning applies to the understanding of how genetic variations affect gene transcription in healthy individuals. A recent GWAS study identified 68 loci associated with platelet count and volume 116. Nine of these genes have transcription factor activity and may regulate genes that affect how platelets are formed.

Finally, there is currently a dedicated drive in the biomedical community to try to produce cells *in vitro* for human use. This includes platelets for transfusion, as it would potentially have major advantages over donor-derived products in terms of safety and blood group matching. The most promising cell source in this aspect is human induced pluripotent stem cells (iPSCs). Protocols to derive MKs from iPSCs have been published, but these clearly fail on two fronts. Firstly, the number of MKs produced per seeded stem cell is very low (an amplification of less than 100 in the best case 117). Secondly, the platelet harvest from these MKs is very often in single figures 118 whilst MKs *in vivo* are estimated to produce 1000 to 2000 platelets per cell. Without an increase in the knowledge of how to promote maturation of MKs from banks of stem cells and of ways to maximize platelet production from these MKs, production of platelets on the scale necessary for a clinically relevant product (each platelet concentrate contains 280 × 10^9^ platelets) will remain a dream.

## Disclosure of Conflict of Interests

The authors state that they have no conflict of interest.

## References

[b1] Krumsiek J, Marr C, Schroeder T, Theis FJ (2011). Hierarchical differentiation of myeloid progenitors is encoded in the transcription factor network. PLoS One.

[b2] Lee TI, Young RA (2000). Transcription of eukaryotic protein-coding genes. Annu Rev Genet.

[b3] Ng RK, Gurdon JB (2008). Epigenetic inheritance of cell differentiation status. Cell Cycle.

[b4] Davidson EH, Erwin DH (2006). Gene regulatory networks and the evolution of animal body plans. Science.

[b5] Doré LC, Crispino JD (2011). Transcription factor networks in erythroid cell and megakaryocyte development. Blood.

[b6] Makeev VJ, Lifanov AP, Nazina AG, Papatsenko DA (2003). Distance preferences in the arrangement of binding motifs and hierarchical levels in organization of transcription regulatory information. Nucleic Acids Res.

[b7] Sanyal A, Lajoie BR, Jain G, Dekker J (2012). The long-range interaction landscape of gene promoters. Nature.

[b8] Marenduzzo D, Faro-Trindade I, Cook PR (2007). What are the molecular ties that maintain genomic loops?. Trends Genet.

[b9] Nolis IK, McKay DJ, Mantouvalou E, Lomvardas S, Merika M, Thanos D (2009). Transcription factors mediate long-range enhancer-promoter interactions. Proc Natl Acad Sci USA.

[b10] Geddis AE (2010). Megakaryopoiesis. Semin Hematol.

[b11] Zheng C, Yang R, Han Z, Zhou B, Liang L, Lu M (2008). TPO-independent megakaryocytopoiesis. Crit Rev Oncol Hematol.

[b12] Malara A, Balduini A (2012). Blood platelet production and morphology. Thromb Res.

[b13] Avecilla ST, Hattori K, Heissig B, Tejada R, Liao F, Shido K, Jin DK, Dias S, Zhang F, Hartman TE, Hackett NR, Crystal RG, Witte L, Hicklin DJ, Bohlen P, Eaton D, Lyden D, Sauvage De F, Rafii S (2004). Chemokine-mediated interaction of hematopoietic progenitors with the bone marrow vascular niche is required for thrombopoiesis. Nat Med.

[b14] Thon JN, Italiano JE (2010). Platelet formation. Semin Hematol.

[b15] Schulze H, Korpal M, Hurov J, Kim S-W, Zhang J, Cantley LC, Graf T, Shivdasani RA (2006). Characterization of the megakaryocyte demarcation membrane system and its role in thrombopoiesis. Blood.

[b16] Italiano JE, Lecine P, Shivdasani RA, Hartwig JH (1999). Blood platelets are assembled principally at the ends of proplatelet processes produced by differentiated megakaryocytes. J Cell Biol.

[b17] Patel SR, Richardson JL, Schulze H, Kahle E, Galjart N, Drabek K, Shivdasani RA, Hartwig JH, Italiano JE (2005). Differential roles of microtubule assembly and sliding in proplatelet formation by megakaryocytes. Blood.

[b18] Italiano JE, Bergmeier W, Tiwari S, Falet H, Hartwig JH, Hoffmeister KM, André P, Wagner DD, Shivdasani RA (2003). Mechanisms and implications of platelet discoid shape. Blood.

[b19] Davis B, Toivio-Kinnucan M, Schuller S, Boudreaux MK (2008). Mutation in beta1-tubulin correlates with macrothrombocytopenia in Cavalier King Charles Spaniels. J Vet Intern Med.

[b20] Kunishima S, Kobayashi R, Itoh TJ, Hamaguchi M, Saito H (2009). Mutation of the beta1-tubulin gene associated with congenital macrothrombocytopenia affecting microtubule assembly. Blood.

[b21] Chang Y, Auradé F, Larbret F, Zhang Y, Couedic Le J-P, Momeux L, Larghero J, Bertoglio J, Louache F, Cramer E, Vainchenker W, Debili N (2007). Proplatelet formation is regulated by the Rho/ROCK pathway. Blood.

[b22] Chen Z, Naveiras O, Balduini A, Mammoto A, Conti MA, Adelstein RS, Ingber D, Daley GQ, Shivdasani RA (2007). The May-Hegglin anomaly gene MYH9 is a negative regulator of platelet biogenesis modulated by the Rho-ROCK pathway. Blood.

[b23] Mazharian A, Wang Y-J, Mori J, Bem D, Finney B, Heising S, Gissen P, White JG, Berndt MC, Gardiner EE, Nieswandt B, Douglas MR, Campbell RD, Watson SP, Senis YA (2012). Mice lacking the ITIM-containing receptor G6b-B exhibit macrothrombocytopenia and aberrant platelet function. Sci Signal.

[b24] Junt T, Schulze H, Chen Z, Massberg S, Goerge T, Krueger A, Wagner DD, Graf T, Italiano JE, Shivdasani RA, Andrian Von UH (2007). Dynamic visualization of thrombopoiesis within bone marrow. Science.

[b25] Kosaki G, Kambayashi J (2011). Thrombocytogenesis by megakaryocyte; interpretation by protoplatelet hypothesis. Proc Jpn Acad, Ser B, Phys Biol Sci.

[b26] Ito T, Ishida Y, Kashiwagi R, Kuriya S (1996). Recombinant human c-Mpl ligand is not a direct stimulator of proplatelet formation in mature human megakaryocytes. Br J Haematol.

[b27] Choi ES, Hokom MM, Chen JL, Skrine J, Faust J, Nichol J, Hunt P (1996). The role of megakaryocyte growth and development factor in terminal stages of thrombopoiesis. Br J Haematol.

[b28] Ogilvy S, Metcalf D, Print CG, Bath ML, Harris AW, Adams JM (1999). Constitutive Bcl-2 expression throughout the hematopoietic compartment affects multiple lineages and enhances progenitor cell survival. Proc Natl Acad Sci USA.

[b29] Bouillet P, Metcalf D, Huang DC, Tarlinton DM, Kay TW, Köntgen F, Adams JM, Strasser A (1999). Proapoptotic Bcl-2 relative Bim required for certain apoptotic responses, leukocyte homeostasis, and to preclude autoimmunity. Science.

[b30] Kaluzhny Y, Yu G, Sun S, Toselli PA, Nieswandt B, Jackson CW, Ravid K (2002). BclxL overexpression in megakaryocytes leads to impaired platelet fragmentation. Blood.

[b31] Clarke MCH, Savill J, Jones DB, Noble BS, Brown SB (2003). Compartmentalized megakaryocyte death generates functional platelets committed to caspase-independent death. J Cell Biol.

[b32] Botton De S, Sabri S, Daugas E, Zermati Y, Guidotti JE, Hermine O, Kroemer G, Vainchenker W, Debili N (2002). Platelet formation is the consequence of caspase activation within megakaryocytes. Blood.

[b33] Josefsson EC, James C, Henley KJ, Debrincat MA, Rogers KL, Dowling MR, White MJ, Kruse EA, Lane RM, Ellis S, Nurden P, Mason KD, O'Reilly LA, Roberts AW, Metcalf D, Huang DCS, Kile BT (2011). Megakaryocytes possess a functional intrinsic apoptosis pathway that must be restrained to survive and produce platelets. J Exp Med.

[b34] White MJ, Schoenwaelder SM, Josefsson EC, Jarman KE, Henley KJ, James C, Debrincat MA, Jackson SP, Huang DCS, Kile BT (2012). Caspase-9 mediates the apoptotic death of megakaryocytes and platelets, but is dispensable for their generation and function. Blood.

[b35] Hamada T, Möhle R, Hesselgesser J, Hoxie J, Nachman RL, Moore MA, Rafii S (1998). Transendothelial migration of megakaryocytes in response to stromal cell-derived factor 1 (SDF-1) enhances platelet formation. J Exp Med.

[b36] Pitchford SC, Lodie T, Rankin SM (2012). VEGFR1 stimulates a CXCR4-dependent translocation of megakaryocytes to the vascular niche, enhancing platelet production in mice. Blood.

[b37] Hoffman R (1989). Regulation of megakaryocytopoiesis. Blood.

[b38] Sabri S, Foudi A, Boukour S, Franc B, Charrier S, Jandrot-Perrus M, Farndale RW, Jalil A, Blundell MP, Cramer EM, Louache F, Debili N, Thrasher AJ, Vainchenker W (2006). Deficiency in the Wiskott-Aldrich protein induces premature proplatelet formation and platelet production in the bone marrow compartment. Blood.

[b39] Mazharian A, Ghevaert C, Zhang L, Massberg S, Watson SP (2011). Dasatinib enhances megakaryocyte differentiation but inhibits platelet formation. Blood.

[b40] Sabri S, Jandrot-Perrus M, Bertoglio J, Farndale RW, Mas VM-D, Debili N, Vainchenker W (2004). Differential regulation of actin stress fiber assembly and proplatelet formation by alpha2beta1 integrin and GPVI in human megakaryocytes. Blood.

[b41] Ghevaert C, Salsmann A, Watkins NA, Schaffner-Reckinger E, Rankin A, Garner SF, Stephens J, Smith GA, Debili N, Vainchenker W, Groot De PG, Huntington JA, Laffan M, Kieffer N, Ouwehand WH (2008). A nonsynonymous SNP in the ITGB3 gene disrupts the conserved membrane-proximal cytoplasmic salt bridge in the alphaIIbbeta3 integrin and cosegregates dominantly with abnormal proplatelet formation and macrothrombocytopenia. Blood.

[b42] Bury L, Malara A, Gresele P, Balduini A (2012). Outside-in signalling generated by a constitutively activated integrin αIIbβ3 impairs proplatelet formation in human megakaryocytes. PLoS ONE.

[b43] Balduini A, Malara A, Pecci A, Badalucco S, Bozzi V, Pallotta I, Noris P, Torti M, Balduini CL (2009). Proplatelet formation in heterozygous Bernard-Soulier syndrome type Bolzano. J Thromb Haemost.

[b44] Golfier S, Kondo S, Schulze T, Takeuchi T, Vassileva G, Achtman AH, Gräler MH, Abbondanzo SJ, Wiekowski M, Kremmer E, Endo Y, Lira SA, Bacon KB, Lipp M (2010). Shaping of terminal megakaryocyte differentiation and proplatelet development by sphingosine-1-phosphate receptor S1P4. FASEB J.

[b45] Zhang L, Orban M, Lorenz M, Barocke V, Braun D, Urtz N, Schulz C, Brühl Von M-L, Tirniceriu A, Gaertner F, Proia RL, Graf T, Bolz S-S, Montanez E, Prinz M, Müller A, Baumgarten Von L, Billich A, Sixt M, Fässler R (2012). A novel role of sphingosine 1-phosphate receptor S1pr1 in mouse thrombopoiesis. J Exp Med.

[b46] Mattia G, Vulcano F, Milazzo L, Barca A, Macioce G, Giampaolo A, Hassan HJ (2002). Different ploidy levels of megakaryocytes generated from peripheral or cord blood CD34 +  cells are correlated with different levels of platelet release. Blood.

[b47] Miyazaki R, Ogata H, Iguchi T, Sogo S, Kushida T, Ito T, Inaba M, Ikehara S, Kobayashi Y (2000). Comparative analyses of megakaryocytes derived from cord blood and bone marrow. Br J Haematol.

[b48] Leysi-Derilou Y, Duchesne C, Garnier A, Pineault N (2012). Single-cell level analysis of megakaryocyte growth and development. Differentiation.

[b49] Jackson CW, Steward SA, Chenaille PJ, Ashmun RA, McDonald TP (1990). An analysis of megakaryocytopoiesis in the C3H mouse: an animal model whose megakaryocytes have 32N as the modal DNA class. Blood.

[b50] Brown AS, Martin JF (1994). The megakaryocyte platelet system and vascular disease. Eur J Clin Invest.

[b51] Raslova H, Roy L (2003). Vourc'h C, Le Couedic JP, Brison O, Metivier D, Feunteun J, Kroemer G, Debili N, Vainchenker W. Megakaryocyte polyploidization is associated with a functional gene amplification. Blood.

[b52] Raslova H, Kauffmann A, Sekkaï D, Ripoche H, Larbret F, Robert T, Roux Le DT, Kroemer G, Debili N, Dessen P, Lazar V, Vainchenker W (2007). Interrelation between polyploidization and megakaryocyte differentiation: a gene profiling approach. Blood.

[b53] Chen Z, Hu M, Shivdasani RA (2007). Expression analysis of primary mouse megakaryocyte differentiation and its application in identifying stage-specific molecular markers and a novel transcriptional target of NF-E2. Blood.

[b54] Shivdasani RA, Rosenblatt MF, Zucker-Franklin D, Jackson CW, Hunt P, Saris CJ, Orkin SH (1995). Transcription factor NF-E2 is required for platelet formation independent of the actions of thrombopoietin/MGDF in megakaryocyte development. Cell.

[b55] Zimmet JM, Toselli P, Ravid K (1998). Cyclin D3 and megakaryocyte development: exploration of a transgenic phenotype. Stem Cells.

[b56] Chang AN, Cantor AB, Fujiwara Y, Lodish MB, Droho S, Crispino JD, Orkin SH (2002). GATA-factor dependence of the multitype zinc-finger protein FOG-1 for its essential role in megakaryopoiesis. Proc Natl Acad Sci USA.

[b57] Shivdasani RA, Fujiwara Y, McDevitt MA, Orkin SH (1997). A lineage-selective knockout establishes the critical role of transcription factor GATA-1 in megakaryocyte growth and platelet development. EMBO J.

[b58] Vyas P, McDevitt MA, Cantor AB, Katz SG, Fujiwara Y, Orkin SH (1999). Different sequence requirements for expression in erythroid and megakaryocytic cells within a regulatory element upstream of the GATA-1 gene. Development.

[b59] Muntean AG, Pang L, Poncz M, Dowdy SF, Blobel GA, Crispino JD (2007). Cyclin D-Cdk4 is regulated by GATA-1 and required for megakaryocyte growth and polyploidization. Blood.

[b60] Ludlow LB, Schick BP, Budarf ML, Driscoll DA, Zackai EH, Cohen A, Konkle BA (1996). Identification of a mutation in a GATA binding site of the platelet glycoprotein Ibbeta promoter resulting in the Bernard-Soulier syndrome. J Biol Chem.

[b61] Ciovacco WA, Raskind WH, Kacena MA (2008). Human phenotypes associated with GATA-1 mutations. Gene.

[b62] Loughran SJ, Kruse EA, Hacking DF, Graaf De CA, Hyland CD, Willson TA, Henley KJ, Ellis S, Voss AK, Metcalf D, Hilton DJ, Alexander WS, Kile BT (2008). The transcription factor Erg is essential for definitive hematopoiesis and the function of adult hematopoietic stem cells. Nat Immunol.

[b63] Stankiewicz MJ, Crispino JD (2009). ETS2 and ERG promote megakaryopoiesis and synergize with alterations in GATA-1 to immortalize hematopoietic progenitor cells. Blood.

[b64] Heller PG, Glembotsky AC, Gandhi MJ, Cummings CL, Pirola CJ, Marta RF, Kornblihtt LI, Drachman JG, Molinas FC (2005). Low Mpl receptor expression in a pedigree with familial platelet disorder with predisposition to acute myelogenous leukemia and a novel AML1 mutation. Blood.

[b65] Sun L, Gorospe JR, Hoffman EP, Rao AK (2007). Decreased platelet expression of myosin regulatory light chain polypeptide (MYL9) and other genes with platelet dysfunction and CBFA2/RUNX1 mutation: insights from platelet expression profiling. J Thromb Haemost.

[b66] Bluteau D, Glembotsky AC, Raimbault A, Balayn N, Gilles L, Rameau P, Nurden P, Alessi MC, Debili N, Vainchenker W, Heller PG, Favier R, Raslova H (2012). Dysmegakaryopoiesis of FPD/AML pedigrees with constitutional RUNX1 mutations is linked to myosin II deregulated expression. Blood.

[b67] Lordier L, Bluteau D, Jalil A, Legrand C, Pan J, Rameau P, Jouni D, Bluteau O, Mercher T, Leon C, Gachet C, Debili N, Vainchenker W, Raslova H, Chang Y (2012). RUNX1-induced silencing of non-muscle myosin heavy chain IIB contributes to megakaryocyte polyploidization. Nat Commun.

[b68] Pang L, Xue H-H, Szalai G, Wang X, Wang Y, Watson DK, Leonard WJ, Blobel GA, Poncz M (2006). Maturation stage-specific regulation of megakaryopoiesis by pointed-domain Ets proteins. Blood.

[b69] Favier R, Jondeau K, Boutard P, Grossfeld P, Reinert P, Jones C, Bertoni F, Cramer EM (2003). Paris-Trousseau syndrome : clinical, hematological, molecular data of ten new cases. Thromb Haemost.

[b70] Raslova H, Komura E, Couédic Le JP, Larbret F, Debili N, Feunteun J, Danos O, Albagli O, Vainchenker W, Favier R (2004). FLI1 monoallelic expression combined with its hemizygous loss underlies Paris-Trousseau/Jacobsen thrombopenia. J Clin Invest.

[b71] Schlaeger TM, Mikkola HKA, Gekas C, Helgadottir HB, Orkin SH (2005). Tie2Cre-mediated gene ablation defines the stem-cell leukemia gene (SCL/tal1)-dependent window during hematopoietic stem-cell development. Blood.

[b72] Chagraoui H, Kassouf M, Banerjee S, Goardon N, Clark K, Atzberger A, Pearce AC, Skoda RC, Ferguson DJP, Watson SP, Vyas P, Porcher C (2011). SCL-mediated regulation of the cell-cycle regulator p21 is critical for murine megakaryopoiesis. Blood.

[b73] Gekas C, Rhodes KE, Gereige LM, Helgadottir H, Ferrari R, Kurdistani SK, Montecino-Rodriguez E, Bassel-Duby R, Olson E, Krivtsov AV, Armstrong S, Orkin SH, Pellegrini M, Mikkola HKA (2009). Mef2C is a lineage-restricted target of Scl/Tal1 and regulates megakaryopoiesis and B-cell homeostasis. Blood.

[b74] Ragu C, Elain G, Mylonas E, Ottolenghi C, Cagnard N, Daegelen D, Passegué E, Vainchenker W, Bernard OA, Penard-Lacronique V (2010). The transcription factor Srf regulates hematopoietic stem cell adhesion. Blood.

[b75] Smith EC, Thon JN, Devine MT, Lin S, Schulz VP, Guo Y, Massaro SA, Halene S, Gallagher P, Italiano JE, Krause DS (2012). MKL1 and MKL2 play redundant and crucial roles in megakaryocyte maturation and platelet formation. Blood.

[b76] Kilbey A, Alzuherri H, McColl J, Calés C, Frampton J, Bartholomew C (2005). The Evi1 proto-oncoprotein blocks endomitosis in megakaryocytes by inhibiting sustained cyclin-dependent kinase 2 catalytic activity. Br J Haematol.

[b77] Shimizu S, Nagasawa T, Katoh O, Komatsu N, Yokota J, Morishita K (2002). EVI1 is expressed in megakaryocyte cell lineage and enforced expression of EVI1 in UT-7/GM cells induces megakaryocyte differentiation. Biochem Biophys Res Commun.

[b78] Maicas M, Vázquez I, Vicente C, García-Sánchez MA, Marcotegui N, Urquiza L, Calasanz MJ, Odero MD (2012). Functional characterization of the promoter region of the human EVI1 gene in acute myeloid leukemia: RUNX1 and ELK1 directly regulate its transcription. Oncogene.

[b79] Albers CA, Paul DS, Schulze H, Freson K, Stephens JC, Smethurst PA, Jolley JD, Cvejic A, Kostadima M, Bertone P, Breuning MH, Debili N, Deloukas P, Favier R, Fiedler J, Hobbs CM, Huang N, Hurles ME, Kiddle G, Krapels I (2012). Compound inheritance of a low-frequency regulatory SNP and a rare null mutation in exon-junction complex subunit RBM8A causes TAR syndrome. Nat Genet.

[b80] Letestu R, Vitrat N, Massé A, Couedic Le JP, Lazar V, Rameau P, Wendling F, Vuillier J, Boutard P, Plouvier E, Plasse M, Favier R, Vainchenker W, Debili N (2000). Existence of a differentiation blockage at the stage of a megakaryocyte precursor in the thrombocytopenia and absent radii (TAR) syndrome. Blood.

[b81] Sultan Y, Scrobohaci ML, Rendu F, Caen JP (1972). Abnormal platelet function, population, and survival-time in a boy with congenital absent radii and thrombocytopenia. Lancet.

[b82] Day HJ, Holmsen H (1972). Platelet adenine nucleotide “storage pool deficiency” in thrombocytopenic absent radii syndrome. JAMA.

[b83] Zahavi J, Gale R, Kakkar VV (1981). Storage pool disease of platelets in an infant with thrombocytopenic absent radii (TAR) syndrome simulating Fanconi's anaemia. Haemostasis.

[b84] Balduini A, Badalucco S, Pugliano MT, Baev D, Silvestri De A, Cattaneo M, Rosti V, Barosi G (2011). In vitro megakaryocyte differentiation and proplatelet formation in Ph-negative classical myeloproliferative neoplasms: distinct patterns in the different clinical phenotypes. PLoS ONE.

[b85] Ghevaert C, Li J, Severin S, Auger J, Watson S, Green AR (2009). Physiological Levels of Jak2 V617F Result in Enhanced Megakaryocyte Differentiation, Proplatelet Formation and Platelet Reactivity. Blood.

[b86] Boissinot M, Lippert E, Girodon F, Dobo I, Fouassier M, Masliah C, Praloran V, Hermouet S (2006). Latent myeloproliferative disorder revealed by the JAK2-V617F mutation and endogenous megakaryocytic colonies in patients with splanchnic vein thrombosis. Blood.

[b87] Stefano De V, Fiorini A, Rossi E, Za T, Farina G, Chiusolo P, Sica S, Leone G (2007). Incidence of the JAK2 V617F mutation among patients with splanchnic or cerebral venous thrombosis and without overt chronic myeloproliferative disorders. J Thromb Haemost.

[b88] Senyuk V, Rinaldi CR, Li D, Cattaneo F, Stojanovic A, Pane F, Du X, Mahmud N, Dickstein J, Nucifora G (2009). Consistent up-regulation of Stat3 Independently of Jak2 mutations in a new murine model of essential thrombocythemia. Cancer Res.

[b89] Séverin S, Ghevaert C, Mazharian A (2010). The mitogen-activated protein kinase signaling pathways: role in megakaryocyte differentiation. J Thromb Haemost.

[b90] Akada H, Akada S, Gajra A, Bair A, Graziano S, Hutchison RE, Mohi G (2012). Efficacy of vorinostat in a murine model of polycythemia vera. Blood.

[b91] Pecquet C, Staerk J, Chaligné R, Goss V, Lee KA, Zhang X, Rush J, Hees Van J, Poirel HA, Scheiff J-M, Vainchenker W, Giraudier S, Polakiewicz RD, Constantinescu SN (2010). Induction of myeloproliferative disorder and myelofibrosis by thrombopoietin receptor W515 mutants is mediated by cytosolic tyrosine 112 of the receptor. Blood.

[b92] Medves S, Noël LA, Montano-Almendras CP, Albu RI, Schoemans H, Constantinescu SN, Demoulin J-B (2011). Multiple oligomerization domains of KANK1-PDGFRβ are required for JAK2-independent hematopoietic cell proliferation and signaling via STAT5 and ERK. Haematologica.

[b93] Desterke C, Bilhou-Nabéra C, Guerton B, Martinaud C, Tonetti C, Clay D, Guglielmelli P, Vannucchi A, Bordessoule D, Hasselbalch H, Dupriez B, Benzoubir N, Bourgeade M-F, Pierre-Louis O, Lazar V, Vainchenker W, Bennaceur-Griscelli A, Gisslinger H, Giraudier S, Bousse-Kerdilès Le M-C (2011). FLT3-mediated p38-MAPK activation participates in the control of megakaryopoiesis in primary myelofibrosis. Cancer Res.

[b94] Raslova H, Baccini V, Loussaief L, Comba B, Larghero J, Debili N, Vainchenker W (2006). Mammalian target of rapamycin (mTOR) regulates both proliferation of megakaryocyte progenitors and late stages of megakaryocyte differentiation. Blood.

[b95] Cornejo MG, Mabialah V, Sykes SM, Khandan T (2011). Lo Celso C, Lopez CK, Rivera-Muñoz P, Rameau P, Tothova Z, Aster JC, DePinho RA, Scadden DT, Gilliland DG, Mercher T. Crosstalk between NOTCH and AKT signaling during murine megakaryocyte lineage specification. Blood.

[b96] Liu Z-J, Italiano J, Ferrer-Marin F, Gutti R, Bailey M, Poterjoy B, Rimsza L, Sola-Visner M (2011). Developmental differences in megakaryocytopoiesis are associated with up-regulated TPO signaling through mTOR and elevated GATA-1 levels in neonatal megakaryocytes. Blood.

[b97] Guglielmelli P, Barosi G, Rambaldi A, Marchioli R, Masciulli A, Tozzi L, Biamonte F, Bartalucci N, Gattoni E, Lupo ML, Finazzi G, Pancrazzi A, Antonioli E, Susini MC, Pieri L, Malevolti E, Usala E, Occhini U, Grossi A, Caglio S (2011). Safety and efficacy of everolimus, a mTOR inhibitor, as single agent in a phase 1/2 study in patients with myelofibrosis. Blood.

[b98] Andrews NC, Erdjument-Bromage H, Davidson MB, Tempst P, Orkin SH (1993). Erythroid transcription factor NF-E2 is a haematopoietic-specific basic-leucine zipper protein. Nature.

[b99] Lecine P, Villeval JL, Vyas P, Swencki B, Xu Y, Shivdasani RA (1998). Mice lacking transcription factor NF-E2 provide in vivo validation of the proplatelet model of thrombocytopoiesis and show a platelet production defect that is intrinsic to megakaryocytes. Blood.

[b100] Fock E, Yan F, Pan S, Chong BH (2008). NF-E2-mediated enhancement of megakaryocytic differentiation and platelet production in vitro and in vivo. Exp Hematol.

[b101] Goerttler PS, Kreutz C, Donauer J, Faller D, Maiwald T, März E, Rumberger B, Sparna T, Schmitt-Gräff A, Wilpert J, Timmer J, Walz G, Pahl HL (2005). Gene expression profiling in polycythaemia vera: overexpression of transcription factor NF-E2. Br J Haematol.

[b102] Wang W, Schwemmers S, Hexner EO, Pahl HL (2010). AML1 is overexpressed in patients with myeloproliferative neoplasms and mediates JAK2V617F-independent overexpression of NF-E2. Blood.

[b103] Lecine P, Italiano JE, Kim SW, Villeval JL, Shivdasani RA (2000). Hematopoietic-specific beta 1 tubulin participates in a pathway of platelet biogenesis dependent on the transcription factor NF-E2. Blood.

[b104] Tiwari S, Italiano JE, Barral DC, Mules EH, Novak EK, Swank RT, Seabra MC, Shivdasani RA (2003). A role for Rab27b in NF-E2-dependent pathways of platelet formation. Blood.

[b105] Kerrigan SW, Gaur M, Murphy RP, Shattil SJ, Leavitt AD (2004). Caspase-12: a developmental link between G-protein-coupled receptors and integrin alphaIIbbeta3 activation. Blood.

[b106] Nagata Y, Yoshikawa J, Hashimoto A, Yamamoto M, Payne AH, Todokoro K (2003). Proplatelet formation of megakaryocytes is triggered by autocrine-synthesized estradiol. Genes Dev.

[b107] Schwer HD, Lecine P, Tiwari S, Italiano JE, Hartwig JH, Shivdasani RA (2001). A lineage-restricted and divergent beta-tubulin isoform is essential for the biogenesis, structure and function of blood platelets. Curr Biol.

[b108] Shiraga M, Ritchie A, Aidoudi S, Baron V, Wilcox D, White G, Ybarrondo B, Murphy G, Leavitt A, Shattil S (1999). Primary megakaryocytes reveal a role for transcription factor NF-E2 in integrin alpha IIb beta 3 signaling. J Cell Biol.

[b109] Bord S, Frith E, Ireland DC, Scott MA, Craig JIO, Compston JE (2004). Estrogen stimulates differentiation of megakaryocytes and modulates their expression of estrogen receptors alpha and beta. J Cell Biochem.

[b110] Fox SW, Chambers TJ (2006). The effect of oestrogen on megakaryocyte differentiation and platelet counts in vivo. Int J Cardiol.

[b111] Takayama M, Fujita R, Suzuki M, Okuyama R, Aiba S, Motohashi H, Yamamoto M (2010). Genetic analysis of hierarchical regulation for Gata1 and NF-E2 p45 gene expression in megakaryopoiesis. Mol Cell Biol.

[b112] McCormack MP, Hall MA, Schoenwaelder SM, Zhao Q, Ellis S, Prentice JA, Clarke AJ, Slater NJ, Salmon JM, Jackson SP, Jane SM, Curtis DJ (2006). A critical role for the transcription factor Scl in platelet production during stress thrombopoiesis. Blood.

[b113] Satoh Y, Matsumura I, Tanaka H, Ezoe S, Fukushima K, Tokunaga M, Yasumi M, Shibayama H, Mizuki M, Era T, Okuda T, Kanakura Y (2008). AML1/RUNX1 works as a negative regulator of c-Mpl in hematopoietic stem cells. J Biol Chem.

[b114] Tripic T, Deng W, Cheng Y, Zhang Y, Vakoc CR, Gregory GD, Hardison RC, Blobel GA (2009). SCL and associated proteins distinguish active from repressive GATA transcription factor complexes. Blood.

[b115] Tijssen MR, Cvejic A, Joshi A, Hannah RL, Ferreira R, Forrai A, Bellissimo DC, Oram SH, Smethurst PA, Wilson NK, Wang X, Ottersbach K, Stemple DL, Green AR, Ouwehand WH, Göttgens B (2011). Genome-wide analysis of simultaneous GATA1/2, RUNX1, FLI1, and SCL binding in megakaryocytes identifies hematopoietic regulators. Dev Cell.

[b116] Gieger C, Radhakrishnan A, Cvejic A, Tang W, Porcu E, Pistis G, Serbanovic-Canic J, Elling U, Goodall AH, Labrune Y, Lopez LM, Mägi R, Meacham S, Okada Y, Pirastu N, Sorice R, Teumer A, Voss K, Zhang W, Ramirez-Solis R (2011). New gene functions in megakaryopoiesis and platelet formation. Nature.

[b117] Lu S-J, Li F, Yin H, Feng Q, Kimbrel EA, Hahm E, Thon JN, Wang W, Italiano JE, Cho J, Lanza R (2011). Platelets generated from human embryonic stem cells are functional in vitro and in the microcirculation of living mice. Cell Res.

[b118] Takayama N, Nishikii H, Usui J, Tsukui H, Sawaguchi A, Hiroyama T, Eto K, Nakauchi H (2008). Generation of functional platelets from human embryonic stem cells in vitro via ES-sacs, VEGF-promoted structures that concentrate hematopoietic progenitors. Blood.

